# Application of Machine Learning to Improve Appropriateness of Treatment in an Orthopaedic Setting of Personalized Medicine

**DOI:** 10.3390/jpm12101706

**Published:** 2022-10-12

**Authors:** Frida Milella, Lorenzo Famiglini, Giuseppe Banfi, Federico Cabitza

**Affiliations:** 1 IRCCS Istituto Ortopedico Galeazzi, Via Cristina Belgioioso 173, 20157 Milano, Italy; 2 DISCo, Dipartimento di Informatica, Sistemistica e Comunicazione, University of Milano–Bicocca, Viale Sarca 336, 20126 Milano, Italy; 3 Faculty of Medicine and Surgery, Università Vita-Salute San Raffaele, 20132 Milano, Italy

**Keywords:** personalized medicine, machine learning, explainable AI, PROMs, class imbalance, assessment tree

## Abstract

The rise of personalized medicine and its remarkable advancements have revealed new requirements for the availability of appropriate medical decision-making models. Computer science is an area that plays an essential role in the field of personalized medicine, where one of the goals is to provide algorithms and tools to extrapolate knowledge and improve the decision-support process. The minimum clinically important difference (MCID) is the smallest change in PROM scores that patients perceive as meaningful. Treatment that does not achieve the minimum level of improvement is considered inappropriate as well as a potential waste of resources. Using the MCID threshold to identify patients who fail to achieve the minimum change in PROM that results in a meaningful outcome may aid in pre-surgical shared decision-making. The decision tree algorithm is a method for extracting valuable information and providing further meaningful information to the domain expert that supports the decision-making. In the present study, different tools based on machine learning were developed. On the one hand, we compared three XGBoost models to predict the non-achievement of the MCID at six months post-operation in the SF-12 physical score. The prediction score threshold was set to 0.75 to provide three decision-making areas on the basis of the high confidence (HC) intervals; the minority class was re-balanced by weighting the positive class to penalize the loss function (XGBoost cost-sensitive), oversampling the minority class (XGBoost with SMOTE), and re-sampling the negative class (XGBoost with undersampling). On the other hand, we modeled the data through a decision tree (assessment tree), based on different complexity levels, to identify the hidden pattern and to provide a new way to understand possible relationships between the gathered features and the several outcomes. The results showed that all the proposed models were effective as binary classifiers, as they showed moderate predictive performance both regarding the minority or positive class (i.e., our targeted patients, those who will not benefit from surgery) and the negative class. The decision tree visualization can be exploited during the patient assessment status to better understand if those patients will benefit or not from the medical intervention. Both of these tools can come in handy for increasing knowledge about the patient’s psychophysical state and for creating an increasingly specialized assessment of the individual patient.

## 1. Introduction

The rise of personalized medicine and its remarkable advancements have revealed new requirements for the availability of appropriate medical decision-making models [1]. Personalized medicine tailors medical intervention based on patient profiles [2]. Personalized treatments, however, are not solely based on biological factors, but should also consider patient perspectives [3], as several studies suggest (e.g., Llamocca et al. [4]). PROMs are validated questionnaires that provide the patients’ subjective assessment of their health status [5] as a direct result of a medical intervention [6]. For a high-value and patient-centered health care system, it is essential to evaluate outcomes from the patient’s point of view [7]. PROMs incorporate the patient-centered assessment of “value” [8] that cannot be measured by traditional outcomes, such as infection rates and readmission rates [5]. The degree to which pre-treatment to post-treatment PROM scores change may provide useful information regarding the value of the intervention [9]. Treatment that does not achieve the minimum level of improvement is considered inappropriate as well as a potential waste of resources. The minimum clinically important difference (MCID) is the smallest change in PROM scores that patients perceive as meaningful [10], and it is used to interpret changes in PROM scores [11]. Using the MCID threshold to identify patients who are unlikely to benefit from treatment, by failing to achieve the minimum change in PROM that results in a meaningful outcome, may aid in pre-surgical shared decision-making [12]. As the MCID has the capability to identify patients as responding to or not responding to a particular therapy based on their own assessment of their health-related status both before and after surgery [13], its use may also contribute to personalized medicine [10].

Computer science is an area that plays an essential role in the field of personalized medicine, where one of the goals is to provide algorithms and tools to extrapolate knowledge and improve the decision-support process. In the present study, different tools based on machine learning were developed, which are helpful in better identifying specific characteristics of a patient and exploiting them for increasingly effective personalized medicine [14]. The goal is twofold: on the one hand, to present the performance of a machine learning model that predicts the non-achievement of the MCID at six months post-operative in the physical score of the 12-item short-form health survey (SF-12) [15]; on the other hand, to model the data through a decision tree (assessment tree) and provide additional knowledge to the physicians, showing them what patterns are possible among the various features in the individual patient. Specifically, this work presents two different decision trees based on complexity levels. The easiest ones are based on the fast and frugal tree algorithm (FFT) [16], which performs quickly with little information because it is designed to be simple in both its computational cost and design. The former is based on the classical implementation of the decision tree algorithm by providing a more detailed but complex data structure.

Both of these tools can come in handy in increasing knowledge about the patient’s psychophysical state and creating an increasingly specialized assessment of the individual patient [17].

### Related Works

Predictive analytics using machine learning techniques is becoming increasingly important in medicine [18], especially in orthopedics [19]. Recent studies have explored the use of PROMs and machine learning (ML) approaches for predicting whether or not patients will experience meaningful improvements (MCID) after hip or knee replacements in the chosen post-surgery outcome measures. For example, Zhang et al. [20] compared four supervised machine learning algorithms (i.e., random forest, extreme gradient boosting, logistic regression with L1-regularization, and support vector machines) and pre-operative PROM thresholds to predict the achievement of MCID for hip and knee osteoarthritis index (Western Ontario and McMaster University Osteoarthritis Index-WOMAC) and for the generic health-related quality of life measures (SF-36 Physical Component Summary (PCS) and Mental Component Summary-MCS) at 24 months following total knee arthroplasty (TKA). Katakam et al. [5] trained five machine learning algorithms (i.e., stochastic gradient boosting, random forest, support vector machines, neural network, and elastic net penalized logistic regression) to predict the MCID’s achievement for the physical functional index of the knee injury and osteoarthritis outcome score (KOOS-PS) within 12 months after total knee arthroplasty. Kunze et al. [21] compared the same set of machine learning algorithms to predict whether patients undergoing total hip arthroplasty would achieve clinically significant improvement 24 months post operatively on the selected outcome measure (i.e., the EuroQoL-VAS). In another study, Huber et al. [22] trained eight supervised machine learning algorithms (i.e., logistic regression, extreme gradient boosting, random forest, multistep adaptive elastic net, neural network, naïve Bayes, k-nearest neighbors, and boosted logistic regression) to predict whether disease-specific and generic post-operative outcomes of total hip and knee replacement surgery (i.e., EQ-5D-3L VAS, Oxford Hip Score (OHS), and Oxford Knee Score -OKS) would improve within 12 months based on the MCID. On the contrary, Fontana et al. [12] compared three machine learning algorithms (i.e., logistic least absolute shrinkage and selection operator, random forest, linear support vector machine) to predict whether patients undergoing total joint arthroplasty would fail to experience clinically significant improvement 24 months post-operative on mixed selected outcome measures (i.e., Knee Disability and Osteoarthritis Outcome Score for joint replacement (KOOS-JR), Hip Disability and Osteoarthritis Outcome Score for joint replacement (HOOS-JR), SF36-PCS and SF36-MCS). Similarly, Harris et al. [23] developed four machine learning algorithms (i.e., logistic regression, logistic least absolute shrinkage and selection operator, gradient boosting machine, and quadratic discriminant analysis) to predict whether patients will not experience clinically significant improvements within 12 months after total knee replacement (i.e., KOOS total, knee injury and osteoarthritis outcome score joint replacement (KOOS-JS), and KOOS subscales).

To the best of our knowledge, few studies focused on predicting whether the minimum clinical improvement (MCID) will not be achieved in the selected post-surgery outcome measures. On the contrary, no studies in the literature exploit the principles of the decision tree for identifying the hidden pattern and providing a new way to understand possible relationships between the gathered features and the several outcomes [24] of the short-form 12 questionnaires.

## 2. Methods

### 2.1. Classification Method

The extreme gradient boosting (XGBoost) algorithm [25] was selected for the classification task. XGBoost is a decision tree ensemble learning algorithm based on the gradient boosting framework [26]. As a random forest is an ensemble of decision trees using the bagging method, XGBoost builds models sequentially to boost the performance of previous models by using gradient descent to minimize errors [27]. XGBoost can be parallelized more efficiently than gradient boosting and incorporates regularization and tree pruning to minimize over-fitting [28]. In addition, XGBoost employs a sparsity-aware algorithm [25] that automatically handles missing data values [29], including hyperparameters that provide tweaking for unbalanced datasets [30]. XGBoost outperforms other algorithms across a wide range of feature sets and in various settings [31], including orthopedics (e.g., Li and Zhang; Bugarin et al. [32,33]).

Concerning the binary classification task, the MCID was computed. The primary aim of the study is to identify early those patients who will not achieve the MCID threshold and will be at risk of not experiencing any clinical improvement due to surgery. It may be beneficial to make better choices during the preoperative stage [12]. The MCID was calculated separately for the SF12 physical score and SF12 mental score, for both hip and knee replacement surgeries, as one-half the standard deviation of the baseline or pre-operative scores, according to the distribution-based approach [34]. Specifically, the thresholds identified for SF12 physical scores were 3.68 and 3.83 for hip and knee, respectively, whereas the values of 5.89 and 5.68 were obtained for SF12 mental scores, in the case of hip and knee, respectively. The overall binary targets (i.e., target for physical and the one for the mental scores) were constructed based on these thresholds such that if the difference between the post-operative score and the pre-operative score was below the aforementioned threshold, the label was set to 1 (indicating no improvement in the PROM score), otherwise 0 (indicating an improvement in the PROM score). Only for the SF12 Physical Score task, the target distribution was significantly impacted by class imbalance. Indeed, the positive class was 21.6% of the total distribution, indicating that patient improvement was the most frequent outcome.

XGBoost algorithm was implemented using Python 3.8.13 and Scikit-learn Python libraries. RStudio 4.2.1 was used for pre-processing steps.

### 2.2. Hyper-Parameter Optimization and Cross-Validation Strategy

The data were partitioned into 85% training (and validation) and 15% test set to evaluate the performance of the developed models.

Random search optimization algorithm was exploited to find the subset of the optimal XGBoost hyperparameters. Random search does not test every possible combination of hyperparameter grid values but only random combinations of a range of values [35]. Only nine hyperparameters were selected in this study, as reported in Table 1. The values for the search space were set taking into account the default values and the effect of these tree-related hyperparameters to prevent overfitting.

Using the same dataset for hyperparameter optimization and model selection can lead to overfitting [36]. Therefore, we used nested-cross validation. Nested-cross validation consists of two loops: inner loop and outer loop. The outer training dataset is split into k-folds inner training and validation datasets (inner loop) where the validation and hyperparameter optimization occur [27]. The outer loop aims at assessing the performance of a method for fitting a model [37], including cross-validation-based hyperparameter tuning: in each iteration of the outer loop, the test set is not used to optimize the model’s performance, which leads to a more accurate selection of the optimal model [37].

### 2.3. Class Imbalance

Predictions resulting from data imbalance issues are generally biased toward the most frequent classes [38]. In this study, class imbalance significantly impacted the target distribution of the physical classification task. Indeed, the positive class was 21.6% of the total distribution, indicating that patient improvement was the most frequent outcome.

The class imbalance is corrected using various sampling strategies, such as re-balancing and cost-sensitive methods (e.g., Tasci et al. [39]). The Synthetic minority oversampling technique (SMOTE) (Chawla et al. [40]), the under-sampling re-balancing strategy and a built-in data balancing method in the XGBoost, the scale_pos_weight [41] were performed. SMOTE is a re-sampling method that oversamples the minority class [42]; the under-sampling strategy reduces the sample size of the majority class [39]; scale_pos_ weight is a hyperparameter that increases the weight of the positive class (i.e., the minority class) [42], hence forcing the model to account for uncommon events by raising the penalty of incorrectly predicting them [43]. Consequently, scale_pos_weight may be used to train a cost-sensitive version of XGBoost for imbalanced classification [44,45]. Scale_pos_weight was set as the ratio of the total number of examples of the negative class over the total number of examples of the positive class [38]. In terms of SMOTE and under-sampling strategy, the minority class and the majority class were re-sampled to provide an equal number of samples to the minority class (majority class) respectively, for the SF12 physical task.

### 2.4. Evaluation Metrics

Various metrics have been used to assess a models’ performances.

In general, accuracy is used to assess binary classifiers’ performance [41]. This measure, however, is useless for imbalance classification tasks [45]. In contrast, recall or sensitivity, the proportion of correctly classified positive examples, is an important metric in imbalanced medical diagnosis because it is entirely dependent on the minority class [45]. Positive predictive value, which is the fraction of samples predicted to be positive that are actually positive, and recall are typically combined to form a single metric known as F-score, which is ananother important metric when dealing with datasets with imbalanced class distributions [45]. F1 score is defined as the harmonic mean of precision and recall [46].

The positive predictive value-recall curve (PR) plots the positive predictive value against the recall [47], while the receiver operating the characteristic curve (ROC) plots the true positive rate (sensitivity, recall) against the false positive rate (1—specificity) [47]. For highly imbalanced datasets, the area under the positive predictive value-recall curve (AUPRC) is a more appropriate metric than the area under the receiver operating characteristic curve (AUROC) [48].

Accurate uncertainty estimation is required to provide adequate support for human decision-making in machine learning models, especially in high-risk settings (e.g., medicine) where an accurate uncertainty estimation is of primary importance [49]. The definition of calibration error is the absolute difference between the mean of the predicted probabilities and the proportion of positive outcomes [50]. The Expected calibration error (ECE) is the weighted average of the calibration errors defined as follows [49]:

$$ECE=\overset{N}{\underset{i=1}{\sum }}P\left(i\right)\;*\left|{\hat{o}}_{i}-e_{i}\right|\;$$

where $e_{i}$ is the average confidence score within *bin i* (i.e., $e_{i}=\frac{1}{\left|S_{i}\right|}\sum_{x\in S_{i}}f\left(x\right)$), ${\hat{o}}_{i}$ is the relative frequency of the positive class in *bin i* (i.e., ${\hat{o}}_{i}=\frac{1}{\left|S_{i}\right|}\sum_{x\in S_{i}}o_{x}$), $P_{i}$ is the proportion of instances that fall within $S_{i}$. In this view, the lower the ECE, the better the calibration of the model.

Therefore, with regard to the binary classification task, we performed the following metrics: the balanced accuracy, i.e., the average of sensitivity and specificity [51]; the balanced sensitivity; the balanced positive predictive value; the balanced F1-score; AUROC, AUPRC, and ECE were also implemented.

### 2.5. Decision Tree for Pattern Analysis & Decision Support

The white-box models can be used for purely predictive purposes and model data patterns [52]; for instance, by applying a decision tree to identify patterns valid for decision support [53]. In this study, we developed a decision tree primarily for two purposes: (1) to identify a possible structure in the data that would facilitate the level of evaluation of the various cases; (2) to identify potential hidden relationships [54]. In addition, the development of the decision tree serves not only as a tool for decision support but also as a means to facilitate the identification of possible variable information from domain experts. Specifically, we sampled data by exploiting the under-sampling approach for the majority class to limit possible model bias [55]. Then, we trained a decision tree based on the following principle: trade-off between overfitting and explainability. Since the application of the model is for pattern discovery purposes only, we purposely overfitted the model to be able to identify in a more granular way the relationships between the various variables. At the same time, we were interested in maintaining a relatively low level model complexity to facilitate feature comprehension and visualization. The hyperparameters setting is reported in Table 2.

The model achieves on average ~77% on all the analyzed metrics (e.g., accuracy, sensitivity). Once the tree was developed, we created two visualizations of the same model, based on the level of complexity and read time. Depending on the split node, it will be necessary to use the representation of that specific subset.

We also developed a simpler decision tree based on the FFT [16] algorithm for the aforementioned reasons. The tree’s construction is based on a few key questions where a binary answer (e.g., yes/no) is returned as output. These questions follow an order based on the level of information contained. The main goal of FFTs is to make decisions in an optimized manner with as few cues as possible. Such models are used especially in contexts where decisions have to be made in a short time. Unlike the decision tree, two subsets of data were used for the development of the FFT: one for the training and one for the test set (the same as those used for XGBoost). During the training phase, the data were sampled following the majority class undersampling approach.

## 3. Results

The study encompassed patients admitted to IRCCS Galeazzi Orthopaedic Institute between January 2013 and February 2022. IRCCS Galeazzi Orthopaedic Institute (IOG) in Milan, Italy, is a teaching hospital specializing in diagnosing and treating of musculoskeletal problems. Approximately 5000 surgeries are performed each year at IOG, which are usually joint (hips and knees) as well as spine-related procedures; we collected PROM data at the IOG via means of computer-assisted telephone-interview or computer-assisted web self-interview both before surgery (pre-operative) and at 6 months after hip and knee surgery.

Data on 3782 patients who had undergone hip arthroplasty and 3024 patients who had undergone knee arthroplasty were extracted from the web-based PRO registry (DataREG) of IOG. The SF-12 scale ranges from 0 (worst possible health condition) to 100 (best possible health condition).

Two records from the hip dataset and one record from the knee dataset were excluded from the analysis. Additionally, 170 patients were excluded because they had not completed both the pre-operative scores and the 6-month follow-up questionnaires for both PROMs of interest (SF12 physical score and SF12 mental score). A total of 3610 patients who had undergone hip replacement surgery and 2911 patients who had undergone knee arthroplasty completed at least one of the baseline or 6-month post-operative scores for the variables of interest. This resulted in a total of 6521 patients being considered for the descriptive analysis.

As reported in Table 3, the majority of the patients had undergone primary unilateral replacements of both hip surgery (89.14%, *n* = 3218) and knee replacement (91.34%, *n* = 2659). However, both unilateral revisions and bilateral arthroplasties were also performed (Table 3). More than half of the patients who had undergone hip replacement were women (55.29%, *n* = 1996); likewise, two-thirds of the sample for knee surgeries were women (67.5%, *n* = 1964). Patients with hip arthroplasty were aged 67.88 ± 11.97 years (mean ± SD). The mean age of the patients with knee replacement was 71.08 ± 8.95. The summaries of ASA scores and length of stay in days are shown recorded in Table 2. Categorical variable was codified as follows: gender (0 female. 1 male).

The percentage of missing values in the instances was used to filter the instances out. Specifically, the observations containing more than 40% missing values were dropped. 1622 cases of hip arthroplasty and 1470 instances related to knee replacement surgeries were missing in the pre-operative or 6 months post-surgery scores for both PROMs of interest (SF12 physical score and SF12 mental score), and they were accordingly dropped. After removing missing values by rows, the features had less than 3% of missing values, excepted for the ASA score (12.21%).

The distribution of the classes was imbalanced. Positive class (minority class) comprised 21.6% of the total distribution for the SF12 physical score task, indicating that patient improvement was the most common outcome. In contrast, the positive class was 61.6% of the total distribution regarding the SF12 mental score task. The study’s primary aim was to develop a machine learning model that finds patients who do not significantly improve within 6 months after surgery. Accordingly, we shed light only on the physical task, where the imbalance data issue may impact the model’s predictive performance due to the minority class.

In total, 2998 instances and 22 features (Table 4) were considered for training and validation.

The XGBoost machine learning algorithm was used as a classifier for training and testing. The XGBoost algorithm with random search optimization procedure was performed threefold.

In the first round, we imputed missing values using the Bayesian ridge estimator of the Python iterative imputer function on the training folds and then on the validation folds. The random search optimization algorithm was applied with a budget of 100 by using the F1 score as model performance evaluation on nested-cross validation (k inner loop = 9, k outer loop = 6). StratifiedkFold cross-validation was used for both loops of the double cross-validation, i.e., inner and outer, to preserve class distributions similar to those in the original data [56]. The XGBoost evaluation metric was set to the default logarithmic loss function, i.e., logloss, a probability-based metric for measuring the performance of classification problems; the scale_pos_weight hyperparameter was set to the ratio of the total number of examples of the negative class over the total number of examples of the positive class to reduce the imbalance bias.

In the second round, the imputation of missing values was performed on the training folds and then applied to the validation folds by using the Bayesian ridge estimator of the iterative imputer function available in Python. The SMOTE technique was applied only to the training folds by re-balancing the minority class, instead of the cost-sensitive approach chosen in the first round. The above-mentioned steps were performed using the pipeline function of Python to chain the steps and prevent data leakage issues. The hyperparameters optimization stage and the XGBoost classifier’s parameters were left unchanged.

In the third round, the Bayesian ridge estimator was trained on the training folds and then executed on the validation folds to handle missing values. Using a common strategy for the imputation of missing values between models enables a fair comparison of the models under development. Furthermore, the majority class was under-sampled in order to provide an equal number of samples to the minority class. As a result of executing the above steps using Python’s pipeline function, data leakage was prevented. The subset of XGBoost’s parameters remained unchanged as well as the hyperparameters tuning phase.

Table 5 shows the hyperparameters identified by the random search optimization procedure for the three models developed.

Models were compared on the test set (i.e., the 15% excluded from training and validation steps) on the basis of the balanced evaluation metrics. The prediction score threshold that divides the classes was set at 0.75 in order to identify only highly confident patients who have not experienced a clinical improvement after surgery (Table 6).

**Figure 1:**
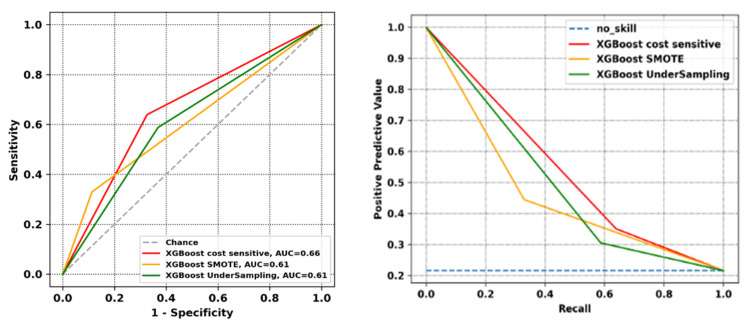
Physical classification task: ROC curve (on the **left**) and positive predictive value-recall curve (on the **right**) (on test set).

Figure 1 reports the ROC curve and the positive predictive value-recall curve for the XGBoost cost sensitive version, XGBoost model with SMOTE over-sampling and XGBoost model with under-sampling approach. The prediction score threshold is conventionally set at 0.5.

**Figure 2:**
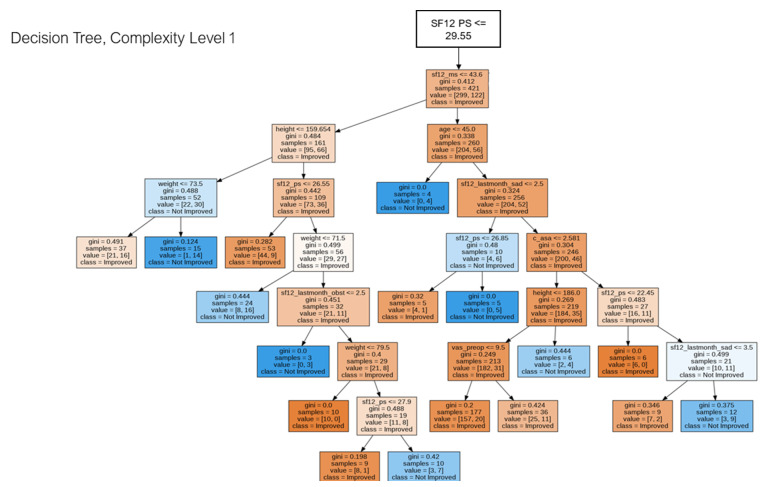
Decision Tree visualization (sub-tree), low complexity.

Figure 2 represents the hidden relationships identified by the decision tree. This sub--tree is referred to the left-hand side of the main decision tree, and it is identified as the tree with the lower readable complexity. For the most complex ones see Appendix A.

**Figure 3:**
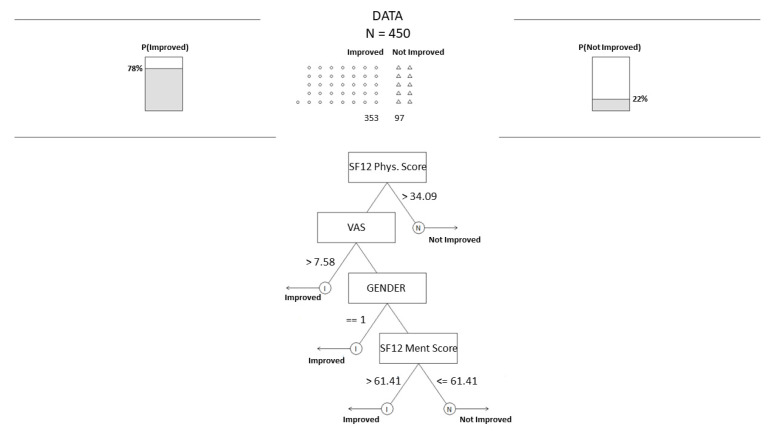
Fast and frugal tree, lowest complexity.

Figure 3 shows the FFT structure, where only a few features were considered. Specifically, only four variables were taken into account: SF12 physical and mental Score, VAS, and Gender. This simple model achieves the following performance on the test set: balanced accuracy 64%, balanced sensitivity 61%, balanced positive predictive value 40%, and AUC 61%.

## 4. Discussion

We developed three XGBoost models with three strategies for coping with the data imbalance issue: an XGBoost model with a built-in data balancing method; an XGBoost model with SMOTE over-sampling technique; and an XGBoost model with an under-sampling technique.

The prediction score threshold that divides the classes was set to 0.75 in order to identify only the patients who would not likely experience clinical improvement after surgery. We adopted this reporting convention and reported only high confidence (HC) accuracy scores to recognize the importance of avoiding false positives and detecting real positives, so as to avoid suggesting inappropriate operations that would not be beneficial for the patients involved. According to this convention, we defined three decision-making areas on the basis of the high confidence (HC) intervals. The rejection area of non-improvement, which includes those patients who will gain from the hip or knee re-placement surgery (i.e., patients who do achieve the MCID threshold); the acceptance area of the target outcome (i.e., the non-improvement), which identifies highly confident patients who will not experience a meaningful improvement after surgery (i.e., patients who do not achieve the MCID threshold); the uncertainty area where the decision making process should be ruled by other criteria, likely to be external.

As shown in Table 6, our results indicate that HC-balanced accuracy was 0.79 [0.71, 0.87], 0.82 [0.71, 0.93], and 0.76 [0.68, 0.84] for the XGBoost cost-sensitive version, XGBoost with SMOTE, and XGBoost model with under-sampling strategy, respectively. The XGBoost model with SMOTE re-balancing option achieved the highest HC-balanced positive predictive value (0.91 [0.83, 0.99]); notwithstanding this, the cost-sensitive version of XGBoost outperforms on the weighted predictive positive value (0.84 [0.77, 0.91]) the XGBoost model with under-sampled majority class (0.80 [0.73, 0.87]). Both the cost-sensitive version of XGBoost and the XGBoost model with SMOTE re-balancing option showed the highest HC-balanced sensitivity (0.84 [0.77, 0.91] and 0.91 [0.83, 0.99], respectively) compared to the XGBoost model with the under-sampling approach, whose average weighted value was 0.75 with an upper bound of 0.83 (0.75 [0.67, 0.83]). Furthermore, the XGBoost model with the under-sampling approach had the lowest HC-F1 score (0.76 [0.68, 0.84]) compared to the other models. Coverage, that is the proportion of cases where the models propose a classification that is relatively low, except for XGBoost under-sampling; however, although this is a limitation of our current study, we believe that this feature should not be considered a shortcoming of the proposed predictive models, but rather a measure to reduce the risk of false alarms and of inducing automation bias. Obviously, these coverage rates should be assessed in real-world settings, by asking human decision makers if they consider receiving appropriateness-related advise for one case out of four or five of small benefit with respect to non-receiving such an advice at all or, rather, a valuable support despite its abstention behaviors for all non-high-confidence cases.

These findings suggest that all the proposed models were effective as binary classifiers, as they showed moderate predictive performance both regarding the minority or positive class (i.e., our targeted patients, those who will not benefit from surgery) and the negative class. Indeed, as shown by the HC-AUROC in Table 6, each one of the developed XGBoost models demonstrates its ability to discriminate between patients who will not perceive an improvement six months post-surgery and those whose health-related status will get better after 6 months. Even more relevant to the purpose of this study, all models showed their ability to handle the minority class (i.e., patients who are unlikely to gain from the surgery). This means that the models are effective in correctly classifying positive examples (i.e., detecting patients who do not improve) and are accurate in predicting the outcome of interest (i.e., avoiding false positives), as reported by HC-AUPRC in Table 5. In particular, the XGBoost model with SMOTE re-balancing option presents the highest HC-AUPRC (0.79 [0.67, 0.91]) compared to XGBoost that is cost-sensitive (0.72 [0.63, 0.81]) and XGBoost with an under-sampled majority class (0.70 [0.62, 0.78]), as shown in Table 6. However, it can only be argued that the XGBoost model with a SMOTE re-balancing strategy would appear to be the best model based on the choices made for the models’ development. Indeed, the models’ performance is inextricably linked to the study setting and the type of (randomized in our case) search used to optimize the hyperparameters. Additionally, the models exhibit calibrated confidence scores, as the HC-1-ECE ranges from 0.86 [0.76, 0.96] to 0.93 [0.88, 0.98]; due to the calibrated results of the models used in this study, the likelihood of the patient’s worsening (our target outcome) is well grounded. Moreover, whenever predictive models produce calibrated probabilities, the more calibrated the probabilities are, the greater the utility expected from the decisions they generate is [50]. Non-calibrated models can have a detrimental effect on healthcare. As a result, we should consider the results of our analysis to be of interest.

ML models’ performance estimates may be negatively affected by imbalanced data, as these models may place disproportionate emphasis on sub-groups with high prevalence (i.e., the proportion of the true negative, which, in our case, is the number of patients whose condition is expected to improve), resulting in an over-estimation of their accuracy. In unbalanced datasets, using AUROC as the sole metric to measure the performance of the models can be misleading, as it may not detect slight changes in the false positive rate and provide overly optimistic results. Indeed, in our study, the HC-AUROC in each model outperforms the related HC-AUPRC, showing the utility of performance estimates unaffected by data imbalance.

Regarding pattern discovery, interesting and possibly helpful information was extracted from the decision tree. For instance, in Figure 2, we can understand how the likely outcome would be if the patient reflects specific feature values. If the SF12 pre-operative physical score is less than 29.55, the height is less than ~156.6 cm, and the weight is more than 73.5 kg, there are high chances of not improving. However, if the weight is less than 73.5 kg, the possibility of improvement from the surgery increases. This information suggests that if the pre-operative physical score is low and the patient is overweight, the patient’s response to the medical intervention could be negative, as shown in the medical literature [57,58,59]. Indeed, it would be of interest to pay more attention to the patient’s dietary habits before the surgery to reduce possible recovery complications [60]. Interestingly, an identified pattern is related to comorbidities. A few patients with a low pre-operative physical score affected by several comorbidities and with an unfortunate emotional state of perceived pain have a lower chance of improvement within the next six months of the surgery.

These examples show how the decision tree visualization can be exploited during the patient assessment status to better understand if those patients will benefit or not from the medical intervention. Moreover, this instrument can be useful for more personalized and precise medicine [61]. In addition, if the domain expert wants to read and understand quickly, FFT can be exploited for this purpose. Indeed, FFTs tend to be resilient against overfitting, speedier and less information-intensive, and simple to understand, use, and convey. By considering the cues, a physician could assess just by looking at the physical score and VAS score (general condition of the patient’s health status). From the FFT, it seems that if the patient has a higher VAS, it starts with a worse state, and if a physical score is lower than 34, there are high chances of improvement after the medical intervention.

## 5. Conclusions

The primary aim of this study is to present the performance of a machine learning model that predicts the non-achievement of the minimal clinically important differences (MCID) in the SF12 physical score six months after surgery. On the other hand, the second aim is to develop a decision tree model (based on several complexity levels) to discover possible important hidden patterns behind the data and develop an informative tool for the decision support systems. The effectiveness of medical therapy should even be grounded on evaluating the changes in PROM scores that patients perceive as meaningful [34]. Indeed, it is crucial to identify early those patients who are unlikely to gain from surgery due to failing to meet the MCID threshold. This may be supported by making more suitable choices in preoperative stages [12] for a more precise and personalized medicine.

The study offers room for further development. First, our study relies on a distribution-based MCID. Future research should also include anchor-based MCID. A recent review by Çelik and colleagues [11] revealed a wide variation in existing estimates of MCIDs using anchor-based techniques in orthopedics across various PROs. However, anchor-based MCID should be able to better reflect changes in outcomes from a patient’s perspective [11]. Moreover, MCID thresholds for different types of surgery should be discussed. Second, this study compares three XGBoost models with three approaches to address the data imbalance issue. While the XGBoost models show moderate and comparable performance, more robust approaches should be considered. Nguyen and Duong [42] compare the predictive performance of resampling methods, such as SMOTE, and cost-sensitive methods, such as focal loss and weighted loss, concluding that methods that adjust the relative cost of error during the training phase are more effective than re-sampling, especially in highly imbalanced data (i.e., ranging from 1% to 5%). Developing a more robust approach to dealing with data imbalance is required even to enhance the effective use of these theoretical solutions as supporting tools in the decision-making process of whether or not medical treatment should be applied. Furthermore, developing a decision tree model, and using it as a method of assessment, may, in some cases, be more practical and more effective in decision support in precision and personalized medicine. It is oriented toward merely explaining the structure and patterns in the data. Supporting humans through machine learning could improve the accuracy and assessment of humans themselves [62]. For instance, assessing the individual patient leads to more and more ad hoc evaluations by limiting possible complications not considered, thanks to the support of machine learning systems.

## Figures and Tables

**Table 1:** Search space for hyperparameters optimization of XGBoost.

	Learning Rate	Min. Child. Weight	Max Depth	Gamma	Colsample Bytree	N. Estimators
Search Space	[0.001, 0.01]	[1, 10]	[3, 20]	[0.01, 1]	[0.01, 1]	[3, 150]

**Table 2:** Hyperparameters setting of decision tree.

Max Depth	Min. Samples Leaf	Min. Samples Split	Max Leaf Nodes
8	2	2	40

**Table 3:** Descriptive statistics.

	HA (*n* = 3610) Number (%)				KA (*n* = 2911) Number (%)			
Unilateral primary	3218 (89.14)				2659 (91.34)			
Unilateral revision	349 (9.67)				213 (7.32)			
Bilateral	43 (1.19)				39 (1.34)			
Gender								
Female	1996 (55.29)				1964 (67.5)			
Male	1614 (44.71)				947 (32.5)			
ASA score								
1	390 (10.83)				131 (4.52)			
2	2326 (64.58)				1854 (63.93)			
3	267 (7.41)				217 (7.48)			
4	1 (0.03)				-			
Missing	618 (17.16)				698 (24.07)			
	Mean	Sd	Min	Max	Mean	Sd	Min	Max
Age (years)	67.88	11.97	14	97	71.08	8.95	33	93
Length of stay (days)	4.86	2.09	0	32	4.72	2.05	0	35

**Table 4:** Selected Features.

	Missing Values (%)
Gender	0
Age	0
ASA score	12.21
VAS total_PreOp	0.97
SF12 Physical ScorePreOp	0
SF12 Mental Score_PreOp	0
BMI height PreOp	2.13
BMI weight PreOp	2.13
SF12 autoevaluation health answer PreOp	0.17
SF12 Score answer PreOp	0.17
SF12 lastmonth resa answer PreOp	0.17
SF12 lastmonth limite answer PreOp	0.17
SF12 lastmonth emo answer PreOp	0.17
SF12 lastmonth ostacolo answer PreOp	0.17
SF12 lastmonth sereno answer PreOp	0.17
SF12 lastmonth energia answer PreOp	0.17
SF12 lastmonth triste answer PreOp	0.17
SF12 lastmonth sociale answer PreOp	0.17
Target phy	0
Operating zone	0
Bilateral Hip	0
Bilateral knee	0

**Table 5:** Hyperparameters identified by the random search optimization procedure of the models.

	Learning Rate	Min. Child. Weight	Max Depth	Gamma	Colsample_bytree	N. Estimators
XGBoost—Cost sensitive	0.171	9	3	0.41	0.91	33
XGBoost—SMOTE	0.051	8	3	0.31	0.51	33
XGBoost—UnderSampling	0.151	5	3	0.51	0.21	63

**Table 6:** The models’ high confidence performance on test set. Binomial confidence intervals are shown at 95% confidence level.

	HC Balanced Accuracy	HC Balanced Sensitivity	HC Balanced F1 Score	HC Balanced PPV	HC AUROC	HC AUPRC	HC 1-ECE	Coverage
XGBoost—Cost sensitive	0.79 [0.71, 0.87]	0.84 [0.77, 0.91]	0.84 [0.77, 0.91]	0.84 [0.77, 0.91]	0.79 [0.71, 0.87]	0.72 [0.63, 0.81]	0.93 [0.88, 0.98]	22%
XGBoost—SMOTE	0.82 [0.71, 0.93]	0.91 [0.83, 0.99]	0.91 [0.83, 0.99]	0.91 [0.83, 0.99]	0.82 [0.71, 0.93]	0.79 [0.67, 0.91]	0.86 [0.76, 0.96]	10%
XGBoost—UnderSampling	0.76 [0.68, 0.84]	0.75 [0.67, 0.83]	0.76 [0.68, 0.84]	0.80 [0.73, 0.87]	0.76 [0.68, 0.84]	0.70 [0.62, 0.78]	0.92 [0.87, 0.97]	25%

**Figure A1:**
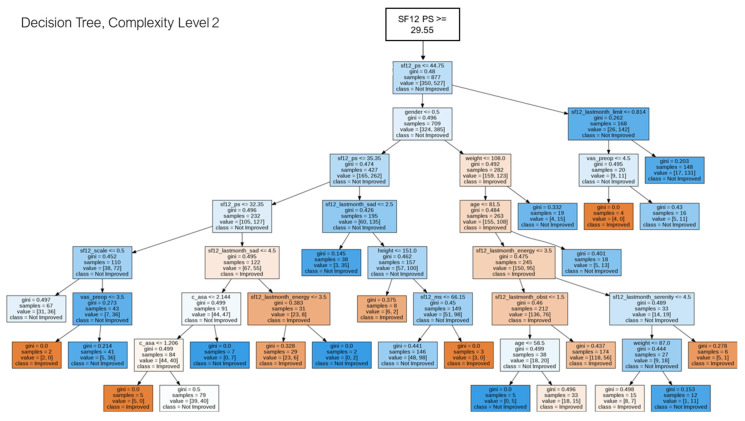
Decision Tree visualization (sub-tree), high complexity.

## Data Availability

The data presented in this study are available in Zenodo at https://doi.org/10.5281/zenodo.7114487 (accessed on 7 October 2022).
